# Unilateral demodicidosis of the nipple/areola complex mimicking nevoid hyperkeratosis

**DOI:** 10.1016/j.jdcr.2024.03.024

**Published:** 2024-04-26

**Authors:** Valentina Caputo, Donata Calò, Franco Rongioletti

**Affiliations:** a Department of Surgical Pathology, ASST Grande Ospedale Metropolitano Niguarda, Milan, Italy; b Department of Multispecialist Medicine, ASST Grande Ospedale Metropolitano Niguarda, Milan, Italy; c Division of Clinical Dermatology, Università Vita-Salute San Raffaele, IRCCS Ospedale San Raffaele, Milano, Italy

**Keywords:** demodicidosis, demodicosis, nevoid hyperkeratosis, nipple

## Introduction

*Demodex* mites, which are common ectoparasites found on the human skin, comprise 2 species: *Demodex folliculorum* and the smaller *Demodex* *brevis*.^1^ These mites inhabit the human pilosebaceous unit and are present in individuals of all ages, except in neonates. Although they are distributed throughout various areas of the skin, they exhibit a preference for the face, particularly the nose and nasolabial folds.

Demodicidosis (demodicosis) is characterized by the overgrowth of *Demodex* mites, leading to a spectrum of dermatological conditions such as papulopustular rosacea, granulomatous rosacea, scalp eruptions, pityriasis folliculorum, spinulosis of the face, hyperpigmented patches, blepharitis, perioral dermatitis, or pustular folliculitis, among other manifestations. There are predisposing factors for the development of the infection such as immunosuppression, diabetes, vasodilatory-related factors, and/or sebaceous hyperplasia.^1^ However, demodectic eruptions may present unexpectedly in various locations and exhibit unusual morphologies.^2^

We report a new presentation of demodicidosis in an immunocompetent female characterized by hyperkeratotic, crusted, hyperpigmented lesions over her areola/nipple complex mimicking nevoid hyperkeratosis of the nipple and areola.

## Case report

**Fig 1:**
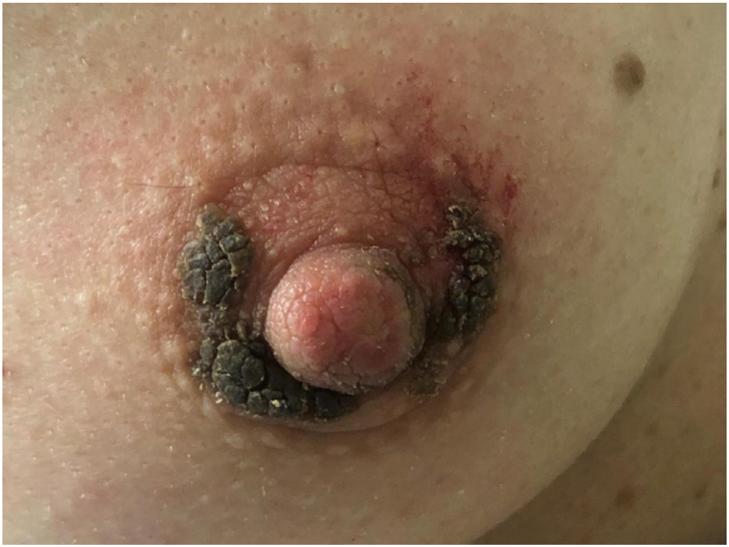
*Brownish* verrucous plaques on left areola, slightly involving also the nipple for 2 months. Some scattered *white* filiform protrusions are visible.

**Fig 2:**
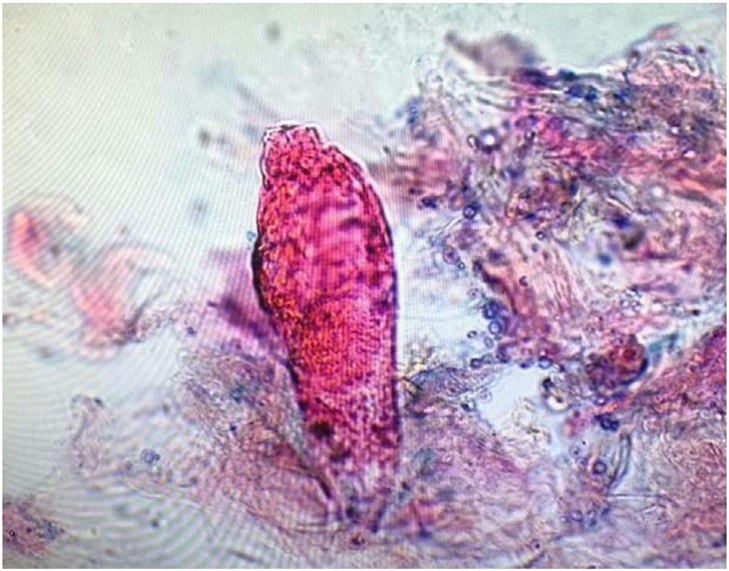
Skin scraping revealed *Demodex folliculorum* mites.

**Fig 3:**
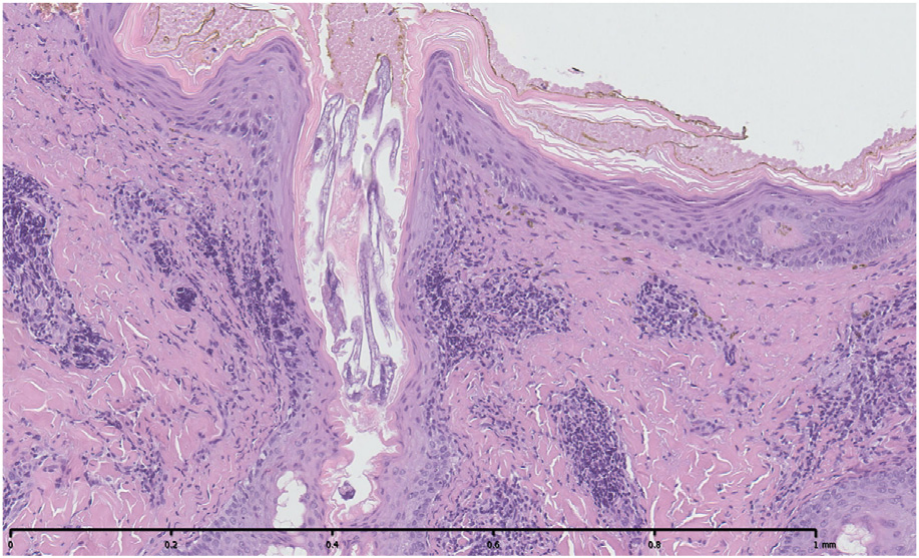
Histopathology showed flat epidermis with overlying thick hyperkeratosis dilated infundibulum with many *Demodex folliculorum* mites and a perivascular and perifollicular lymphocytic infiltrate with some neutrophils in the superficial dermis.

**Fig 4:**
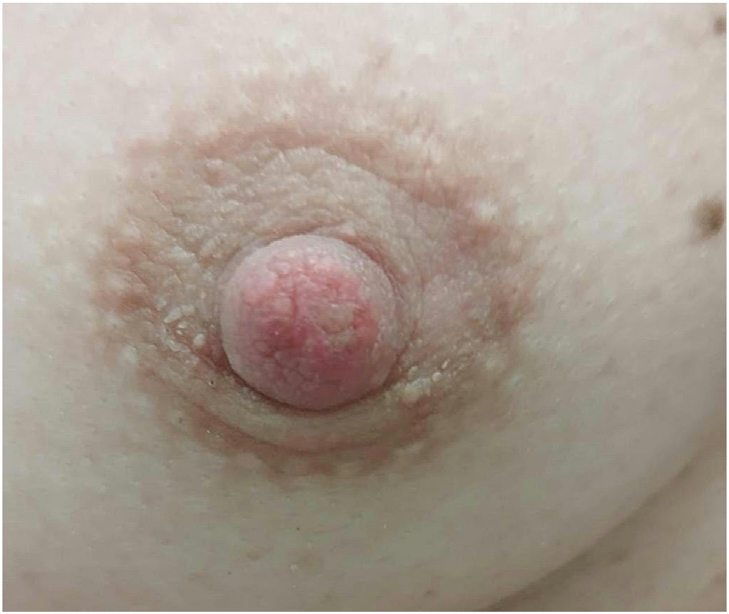
Topical treatment with 1% ivermectin cream twice a day with 10% urea led to complete clearing of skin lesions after 4 weeks.

A 39-year-old healthy white woman presented with a 6-month history of growths on her left areola and nipple. She referred slight pruritus but denied associated pain and discharge. The patient was immunocompetent and was not taking any medications. Physical examination revealed brownish verrucous plaques on her left areola, slightly involving also the nipple (Fig 1). The remainder of the skin surface including the face was normal. After noting some scattered white filiform protrusions from the plaque (Fig 1), skin scraping was performed revealing *D folliculorum* mites (Fig 2). Histological examination showed flat epidermis with overlying thick hyperkeratosis. Within the dilated infundibulum of the hair follicle many *D folliculorum* mites were found (Fig 3). A perivascular and perifollicular lymphocytic infiltrate with some neutrophils in the superficial dermis was observed. Neither bacteria nor fungi were observed with Giemsa and periodic-acid-schiff stain. Topical treatment with 1% ivermectin cream twice a day with 10% urea led to complete clearing of skin lesions after 4 weeks (Fig 4). At the end of treatment, no *Demodex* mites could be demonstrated by scraping from the areola. No recurrence has been observed after a 2-month follow-up.

## Discussion

Although *Demodex* mites are commonly found throughout various regions of the skin, their specific role in dermatological conditions, especially in the nipple/areola area, requires further investigation. The initial documentation of *Demodex* presence in nipple examinations dates back to 1946 when Garven observed their existence in 10 out of 13 nipples, noting a correlation with chronic inflammation around the sebaceous follicle and raising questions about their potential contribution to nipple soreness.^3^

Since Garven’s first report, attention to the presence of *Demodex* mites in the nipple has been sporadic. A prospective study involving adult autopsies found these ectoparasites in 41.4% (58 out of 140) of nipples, with a notably higher frequency in males.^4^ Fidler, examining routine single sections from 50 mastectomy specimens, identified *Demodex* mites in 15 nipples, primarily within hair follicles or sebaceous ducts rather than nipple ducts.^5^ Some cases showed the presence of chronic inflammatory cells near the mites, although no significant pathological changes were observed in hair follicles or sebaceous glands.

As for anecdotal cases of demodicidosis affecting the nipple/areola, Jansen et al reported a woman with unilateral eczema-like features on the nipple, responding to 5% topical permethrin.^6^ Another instance involved bilateral demodicosis of the nipple-areola complex in an immunocompetent woman with bilateral eczema-like lesions, which responded to topical 1% ivermectin.^7^ Additional reports included a patient with itching in both nipples without visible lesions.^8^ In a separate case, a 56-year-old woman had *D folliculorum* identified solely from her milky, white, gelatinous nipple discharge, diagnosed through cytology.^9^ Finally, a 54-year-old woman initially suspected of having Paget disease of the nipple was found to have a substantial presence of *Demodex* within the follicles upon histopathological examination.^10^

The pathogenic mechanism of *Demodex* mites causing clinical manifestations in the nipple/areola complex remains elusive. Unlike previously reported cases, our patient's presentation is unique, resembling nevoid hyperkeratosis of the nipple and areola. Nevoid hyperkeratosis is a rare, acquired, benign condition with diffuse, hyperpigmented, verrucous plaques on the nipple and/or areola, typically affecting women in the second or third decade. Histological changes are nonspecific, resembling those in epidermal nevus and acanthosis nigricans. Treatment is generally unsatisfactory, and the condition tends to persist without therapy. Seborrheic keratosis could also be taken into consideration, but histopathology differed significantly, and it did not respond to antiparasitic treatment.

Despite the absence of local or systemic risk factors indicative of *Demodex* infestation, the identified overgrowth of *D folliculorum* through various diagnostic methods such as skin scraping and histopathology and the positive response to topical ivermectin therapy suggest a causative role of *Demodex* in our patient. These cases collectively underscore the diverse clinical presentations of demodicidosis affecting the nipple-areola complex, ranging from itching to eczema-like or Paget-like skin lesions to an unusual scenario mimicking nevoid hyperkeratosis or seborrheic keratosis. They emphasize the diagnostic challenges, the importance of considering *Demodex* in the differential diagnosis, and the efficacy of topical treatments such as permethrin and ivermectin. This presentation also highlights the need for further research to elucidate the nuanced role of *Demodex* mites in various dermatological manifestations.

## Conflicts of interest

None disclosed.
